# Daytime Variation in Serum Progesterone During the Mid-Luteal Phase in Women Undergoing *In Vitro* Fertilization Treatment

**DOI:** 10.3389/fendo.2018.00092

**Published:** 2018-03-19

**Authors:** Lise Haaber Thomsen, Ulrik Schiøler Kesmodel, Claus Yding Andersen, Peter Humaidan

**Affiliations:** ^1^The Fertility Clinic, Skive Regional Hospital, Skive, Denmark; ^2^Department of Clinical Medicine, Aarhus University, Aarhus, Denmark; ^3^The Fertility Clinic, Herlev Hospital, Herlev, Denmark; ^4^Department of Clinical Medicine, University of Copenhagen, Copenhagen, Denmark; ^5^Laboratory of Reproductive Biology, The Juliane Marie Centre for Women, Children and Reproduction, University Hospital of Copenhagen, University of Copenhagen, Copenhagen, Denmark

**Keywords:** serum progesterone, *in vitro* fertilization, serum estradiol, luteal phase, daytime variation

## Abstract

**Objective:**

To investigate whether mid-luteal serum progesterone (P_4_) exhibits significant fluctuations during a 12-h daytime period in women undergoing *in vitro* fertilization (IVF) and to explore whether the extent of these fluctuations could impact the interpretation of luteal progesterone levels in a clinical setting.

**Design:**

Explorative pilot study.

**Setting:**

Public hospital-based fertility unit.

**Patients:**

Ten women undergoing IVF treatment.

**Intervention:**

Seven days after oocyte pick-up, patients underwent frequent repeated blood sampling (every 60 min for 12 h and during two of these hours, every 15 min). Serum samples were analyzed for progesterone, estradiol, and luteinizing hormone (LH).

**Main outcome measures:**

Daytime fluctuations in s-progesterone and s-estradiol.

**Results:**

There was a significant positive correlation between median P_4_ levels and the magnitude of P_4_ variations—women with median P_4_ < 60 nmol/l had clinically stable P_4_ levels throughout the day, while patients with median P_4_ > 250 nmol/l exhibited periodic P_4_ peaks of several hundred nanomoles per liter. These endogenous P_4_ fluctuations were observed irrespective of the type of stimulation protocol or mode of triggering of final oocyte maturation and despite the fact that LH was under the detection limit at the time of measurement. Simultaneously, large fluctuations were seen in s-estradiol.

**Conclusion:**

Monitoring of early to mid-luteal P_4_ levels in IVF cycles may be valuable in the planning of individualized luteal phase support in the attempt to increase reproductive outcomes. The prerequisite for luteal phase monitoring is, however, that the validity of a single measured P_4_ value is reliable. We show for the first time, that a single P_4_ measurement in the low progesterone patient quite accurately reflects the corpus luteum function and that the measurement can be used to detect IVF patients with a need of additional exogenous luteal P_4_ administration.

## Introduction

The human corpus luteum (CL) is a transient ovarian endocrine gland, which is active during the luteal phase of the menstrual cycle and in early pregnancy until gestational week 8. The CL produces significant amounts of progesterone (P_4_), estradiol (E_2_), and androgens as well as growth factors and nonsteroidal hormones. The overall maintenance of CL function is critically dependent upon regular stimulation of pituitary luteinizing hormone (LH) or human chorionic gonadotropin (hCG) to sustain the steroidogenesis from the luteinized granulosa and theca cells (1). A sufficient P_4_ production from the CL is an absolute necessity for the decidualization of the endometrium preceding implantation and the establishment of early pregnancy. Progesterone secretion from the CL is maximal during the mid-luteal phase inducing a serum P_4_ level of approximately 40–60 nmol/l in the natural cycle (2, 3).

During ovarian stimulation for *in vitro* fertilization (IVF) supra-physiological levels of E_2_ are obtained during the late follicular phase as a result of the multifollicular growth. This hyper-estrogenic state must be counterbalanced in the luteal phase by an increased P_4_ load to achieve a receptive endometrium in time for embryo transfer. Previously, Humaidan and co-workers showed that the use of GnRH agonist trigger in IVF cycles followed by a standard vaginal luteal phase support resulted in mid-luteal P_4_ levels comparable to levels seen in the natural cycle (39 nmol/l) (4). However, in contrast to what was expected, this P_4_ level was too low to secure successful implantation and pregnancy, resulting in an ongoing IVF pregnancy rate of only 6%. Thus, emphasizing the fact that the P_4_ requirement during the luteal phase of the stimulated cycle is greater than that of the natural cycle. When the luteal phase support was modified by adding a bolus of 1,500 IU hCG on the day of oocyte retrieval, the mid-luteal P_4_ level of the GnRHa triggered cycle increased to 74 nmol/l resulting in a delivery rate of 24% per transfer (5). It seems that a mid-luteal serum P_4_ threshold of approximately 80–100 nmol/l exists after IVF treatment followed by fresh embryo transfer, and that this threshold must be surpassed in order to secure a successful reproductive outcome (6). The traditional luteal phase support in artificial IVF cycles with administration of vaginal micronized P_4_ induces a luteal serum P_4_ level of approximately 40 nmol/l (7–9). Thus, a substantial additional endogenous P_4_ production by the CL is mandatory to surpass the P_4_ threshold to subsequently optimize the chance of pregnancy following IVF treatment. Traditionally, clinicians do not monitor the luteal phase P_4_ levels in the firm belief that the luteal phase support will cover the P_4_ need of the cycle. However, we have previously seen that more than 25% of IVF patients in both the hCG and GnRHa triggered group have a mid-luteal serum P_4_ below 60 nmol/l despite luteal phase support and the fact that they had more than 14 follicles on the day of aspiration (10). Furthermore, data from non-human species (11, 12) and data from human frozen/thawed embryo cycles (13, 14) have shown that an optimal luteal P_4_ range exists and that pregnancy outcome is reduced not only below but also above this optimal P_4_ level. Whether this is also the case following IVF and fresh embryo transfer, is still to be explored. If this is the case, monitoring of luteal P_4_ levels may help to improve the reproductive outcome in IVF cycles by allowing an individualization of treatment based on the serum P_4_ measurements.

However, mid-luteal P_4_ measurements are complicated by the pulsatile nature of hormone secretion from the CL. Filicori and co-workers (15) showed that plasma P_4_ concentrations exhibit large and rapid fluctuations during the mid-luteal phase of naturally cycling women. Thus, P_4_ levels ranged from values as low as 7 nmol/l to peaks of 128 nmol/l within minutes during a 24-h study period. In the natural cycle, two distinguishable types of P_4_ pulses exist during the mid-luteal phase: those preceded by an LH pulse and others emerging at time of LH quiescence; the latter being a result of an autonomous steroid secretion by the CL independent of LH activity. During the mid-luteal phase of the stimulated IVF cycle, the pituitary is suppressed by the negative feedback from supra-physiological steroid levels and s-LH is significantly reduced to levels much lower (0.5–0.7 IU/l) than seen in the mid-luteal phase of the natural cycle (5–7 IU/l) (16–18). How this diminished LH pulse activity influences the secretory pattern of ovarian steroidogenesis during the mid-luteal phase of an IVF cycle is until now unknown.

The present study was performed to explore whether mid-luteal serum P_4_ levels in an IVF cycle exhibit a similar high-pulsatile pattern as seen during the natural cycle, knowing that the LH pulse activity is distinctly reduced. From a clinical point of view, we wanted to investigate whether a single morning P_4_ measurement provided a reliable index of mid-luteal CL function following IVF treatment.

## Materials and Methods

### Study Population

Ten female patients undergoing IVF/ICSI at the Fertility Clinic in Skive, Denmark, from December 2014 to December 2015 volunteered to participate in the study. Clinical information regarding age, body mass index (BMI), smoking habits, biochemical reproductive profile, cause of infertility, prior IVF attempts, course of stimulation, and laboratory results were recorded. Baseline characteristics of participants are provided in Table 1. Written informed consent was obtained from all patients prior to study participation. Participants were chosen so as to represent both the long GnRH agonist cycle as well the GnRH antagonist cycle and different types of triggering for final oocyte maturation (hCG or GnRH agonist).

**Table 1:** Description of demographic data, ovarian stimulation, luteal phase support, and progesterone levels in study patients.

Patient	Age (years)	Body mass index (kg/m^2^)	Basal FSH (IU/l)	Cause of infertility	Protocol	Total FSH sum (IU)	Duration of FSH stimulation (days)	Ovulation trigger	Luteal phase support	No. of follicles > 14 mm	No. of oocytes	No. of MII	Mid-luteal P_4_ (nmol/l)
1	38	24.4	6.5	Male factor	Long GnRHa	3,225	11	hCG 10,000 IU	Lutinus 300 mg daily	5	5	1	89
2	37	22.8	6.6	No male partner/endometriosis	GnRH antagonist	1,725	9	Suprefact 0.5 mg	Lutinus 300 mg daily + 1,500 hCG (OPU) + 1,000 hCG (OPU + 5)	10	11	10	283
3	39	30.0	6.4	No male partner	Long GnRHa	3,450	12	Ovitrelle 6,500 IU	Lutinus 300 mg daily	14	12	12	277
4	34	22.2	14.7	Unexplained	Long GnRHa	3,600	12	hCG 10,000 IU	Lutinus 300 mg daily	9	8	8	213
5	28	19.9	5.9	Male factor	Long GnRHa	2,925	11	hCG 10,000 IU	Lutinus 300 mg daily	9	6	3	97
6	37	25.3	8.3	Unexplained	Long GnRHa	3,600	12	Ovitrelle 6,500 IU	Lutinus 300 mg daily	11	8	8	376
7	28	31.8	3.9	Endometriosis	GnRH antagonist	2,025	9	Suprefact 0.5 mg	Crinone 180 daily + 1,500 hCG (OPU)	17	11	11	36
8	36	25.6	4.9	Tubal factor	GnRH antagonist	1,913	9	Suprefact 0.5 mg	Crinone 180 daily + 1,500 hCG (OPU)	19	13	13	55
9	40	34.5	7.2	Unexplained	Long GnRHa	2,250	9	hCG 10,000 IU	Lutinus 300 mg daily	7	6	6	54
10	36	29.4	7.2	Male factor	GnRH antagonist	3,938	15	Suprefact 0.5 mg	Lutinus 300 mg daily + 1,000 hCG (OPU) + 500 (OPU + 5)	20	14	8	161

*Mid-luteal P_4_ = median progesterone level (nmol/l) 7 days after oocyte retrieval*.

*hCG, human chorionic gonadotropin; OPU, oocyte pick-up*.

### Protocols for Ovarian Stimulation

Six patients were treated in a long GnRH agonist cycle with pituitary suppression using SC injection of Buserelin 0.8 mg (Suprefact^®^; Sanofi, Denmark) starting in the mid-luteal phase of the preceding cycle. A daily dose of 0.4 mg Buserelin was administered until the day before ovulation triggering. On day 2 of the cycle, a transvaginal ultrasound examination was carried out, and in case of an endometrial thickness < 4 mm, ovarian stimulation started with corifollitropin-alfa (Elonva^®^; MSD, Denmark) in combination with either r-FSH/rLH (Pergoveris^®^; Merck Biopharma, Denmark) or hMG (Menopur^®^, Ferring Pharmaceuticals, Denmark). The gonadotropin dosage was determined individually based on patient age, BMI, baseline FSH, previous response to gonadotropins, and antral follicle count and adjusted by monitoring follicular size by transvaginal ultrasound during treatment. Final oocyte maturation was induced with either hCG 10,000 IU SC (Pregnyl^®^, MSD, Denmark) or 6,500 IU SC (Ovitrelle^®^, Merck Biopharma, Denmark) when two or more leading follicles reached a mean diameter of 17 mm. Oocyte retrieval was carried out 36 h after hCG administration. IVF/ICSI procedures and embryo culture were performed according to normal clinical practice. A maximum of two embryos were transfered on day 3 or day 5 after oocyte retrieval. Luteal phase support was given as vaginal micronized P_4_ (Lutinus^®^ 300 mg daily, Ferring Pharmaceutical, Denmark or Crinone^®^ 180 mg daily, Merck Biopharma, Denmark) starting 1 day after oocyte pick-up (OPU).

In four patients the GnRH antagonist protocol was used. On day 2 of the cycle ovarian stimulation commenced with either r-FSH (Gonal-F^®^; Merck Biopharma, Denmark) or hMG (Menopur^®^, Ferring Pharmaceuticals, Denmark) after a vaginal ultrasound examination. Daily GnRH antagonist co-treatment (Orgalutran^®^ 0.25 mg/day, MSD, Denmark) was added at a follicle size of 12 mm. The FSH dose was individually adjusted according to the ovarian response. Final oocyte maturation was induced with SC Buserelin 0.5 mg (Suprefact^®^; Sanofi, Denmark) as soon as two or more follicles of ≥17 mm were present. Oocyte retrieval was carried out 36 h later. A maximum of two embryos were transferred on day 3 or day 5 after OPU. Luteal phase support was given in an individualized regimen consisting of vaginal administration of 300 mg micronized P_4_ daily (Lutinus^®^, Ferring Pharmaceuticals, Denmark) in combination with a bolus of hCG (1,000–1,500 IU) on the day of oocyte retrieval (5, 10). Based on the individual ovarian response to stimulation, some patients received an additional hCG bolus on OPU + 5 (500–1,000 IU) (10). See Table 1 for details. Vaginal P_4_ administration continued until the day of pregnancy testing (hCG trigger) or until seventh gestational week (GnRHa trigger).

### Blood Sampling

Blood sampling was conducted during the mid-luteal phase, i.e., 7 days after OPU (OPU + 7). Patients were admitted to the fertility unit early in the morning and stayed at the clinic for the subsequent 12 h. The starting time for blood sampling was between 6 a.m. and 9 a.m. for all patients. Participants were allowed normal daily life activities during the study period.

An intravenous cannula was inserted into a vein in the antecubital fossa and blood samples (4 ml) were drawn every 60 min for 12 h (*n* = 10) and for two of these hours every 15 min (*n* = 8 because of difficult venous access in two patients). After coagulation at room temperature, blood samples were centrifuged and serum was isolated and stored at −80°C until analysis.

### Hormone Measurement

Serum P_4_ (nmol/l), E_2_ (pmol/l), and LH (IU/l) concentrations were measured using automated electro chemiluminescent immunoassays (Cobas^®^ Modular analytics E170, Roche Diagnostics, Switzerland) routinely used for analysis at Department of Biochemistry, Viborg Regional Hospital, Denmark. All measurements were performed according to manufacturer’s instructions using a commercially available chemiluminescent immunoassay kit intended for measurements in serum.

The detection limit of hormones was 0.2 nmol/l, 18.4 pmol/l, and 0.1 IU/l for P_4_, E_2_, and LH, respectively. All serum samples from each patient were measured within the same assay run. All hormone concentrations above the assay detection limit were measured in duplicate. The intra-assay coefficients of variation for P_4_, E_2_, and LH were all below 4%.

### Statistics

Data are presented as mean ± SD or median and range when appropriate. The maximum absolute variation (MAV) in serum P_4_ over a 12-h period is given as the maximum P_4_ concentration − minimum P_4_ concentration during the time of sampling for each patient.

Spearman’s correlation coefficient (*r*) was calculated to correlate median steroid levels with the maximum absolute hormone variation during the day (MAV). A *p* value < 0.05 was considered to be statistically significant. All analyses were performed using STATA, version 13.

### Ethics

The study was conducted according to the declaration of Helsinki for Medical Research and approved by the local Ethics Committee of the Central Denmark Region. ClinicalTrial.gov registration number NCT02673034.

## Results

### Patient Characteristics

Patients had a mean age of 35.3 ± 4.2 years, mean BMI of 26.6 ± 4.7 kg/m^2^ and 1.9 ± 2.0 prior IVF attempts. Median level of FSH for all patients was 6.55 UI/l (interquartile range 5.9;7.2 IU/l). All participants were non-smokers. In four patients the cause of infertility was non-female (male factor or no male partner), in three patients the cause was female (tubal factor or endometriosis) and in three patients the cause of infertility was idiopathic (unexplained). See Table 1 for details.

### Overall Mid-Luteal Progesterone Values

Three patients had median mid-luteal P_4_ levels below 60 nmol/l. Two of these patients (#7 and #8) were triggered with a GnRH agonist and received luteal phase support with 1,500 IU hCG (OPU) and vaginal P_4_ (Crinone 180 mg daily). Despite having 19 and 17 follicles ≥ 14 mm and 11–13 mature oocytes retrieved at the day of OPU they presented with a mid-luteal P_4_ of only 55 and 36 nmol/l, respectively. The other patient (#9) with P_4_ < 60 nmol/l was triggered with 10,000 IU hCG and had seven follicles > 14 mm at the day of aspiration.

### Overall Mid-Luteal LH Values

None of the patients downregulated in a long GnRH agonist protocol (*n* = 6) had s-LH levels above the detection limit of the assay at any point during measurement (i.e., LH < 0.1 IU/l). In three of the four patients stimulated in the GnRH antagonist protocol a modest LH pulse activity was seen with LH amplitudes ranging from 0.2 to 2.8 UI/l. In all patients, the LH peak was followed by an increase in serum P_4_ ranging from 4 to 36 nmol/l.

### Daytime Variation in Serum Progesterone

**Figure 1:**
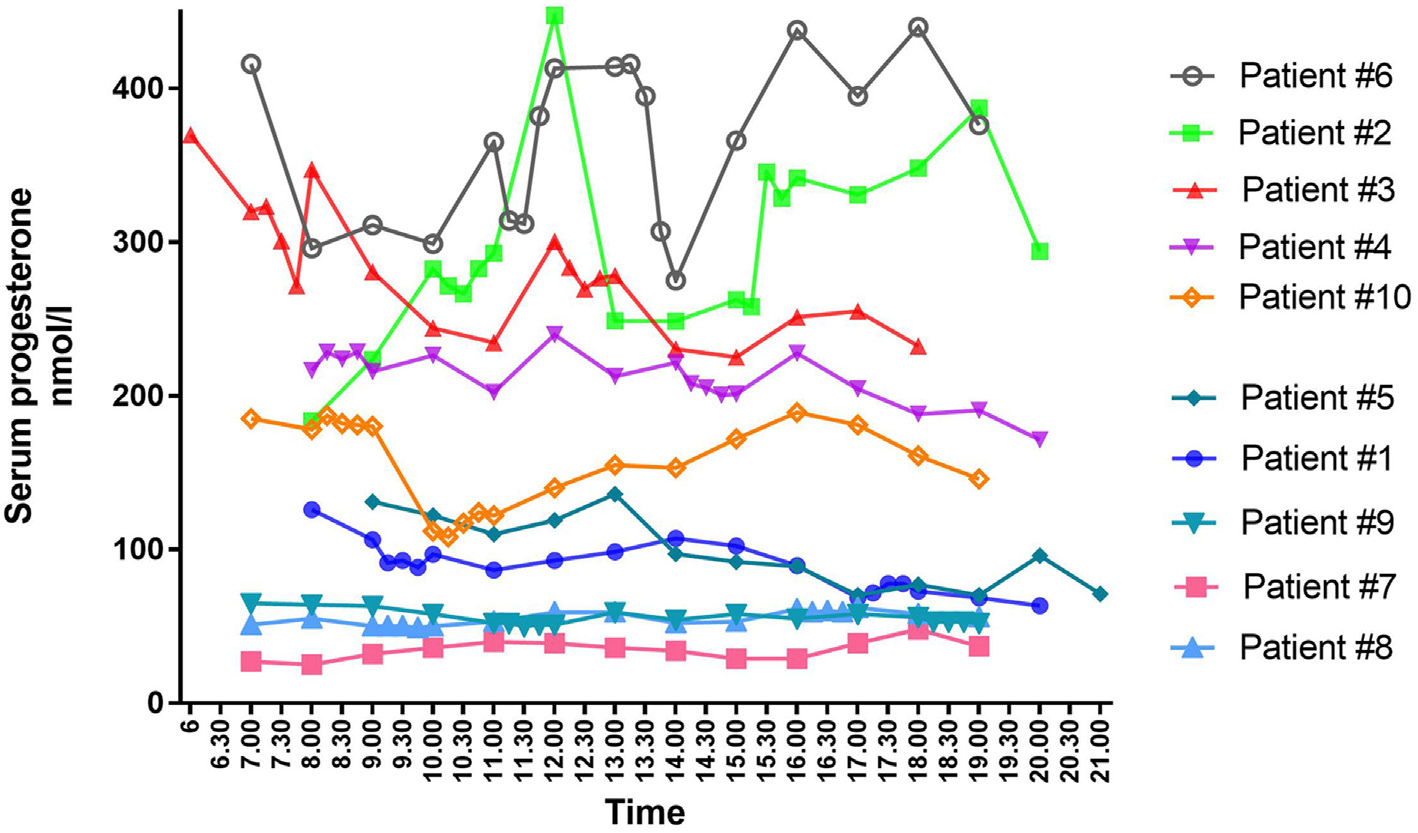
Individual mid-luteal serum profiles of progesterone over a 12-h interval in 10 patients undergoing controlled ovarian stimulation for *in vitro* fertilization treatment.

As seen during the natural cycle, large fluctuations in mid-luteal P_4_ were also present during daytime in some of the women undergoing IVF treatment (Figure 1). Fluctuations in luteal steroids were seen independent of the choice of stimulation protocol, the mode of final oocyte maturation and the type of luteal phase support.

**Figure 2:**
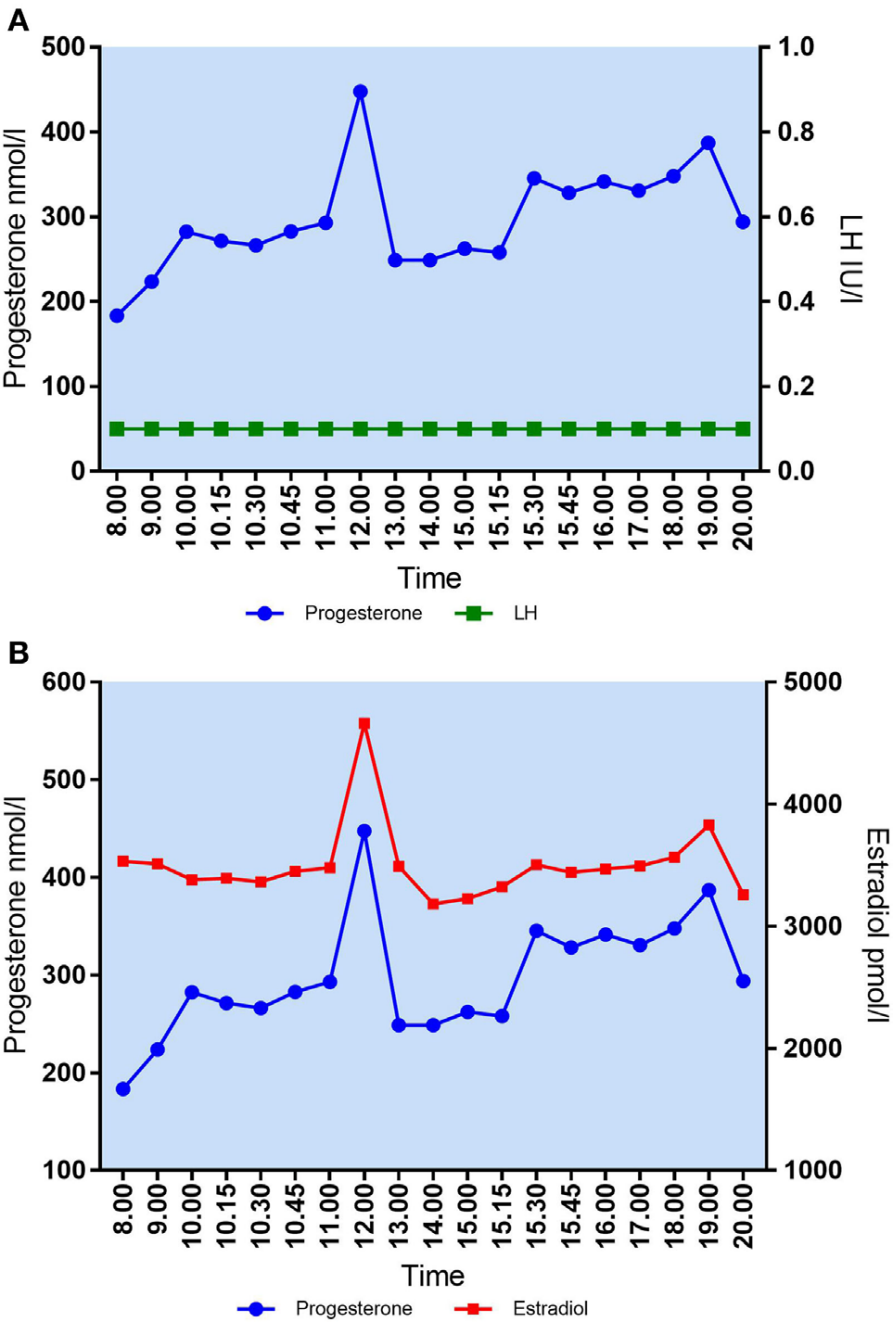
**(A)** Daytime variation in mid-luteal s-progesterone and s-LH in patient #2. Median P_4_ = 283 nmol/l. Maximal variation P_4_/12 h = 265 nmol/l. **(B)** Daytime variation in mid-luteal s-progesterone and s-estradiol in patient #2. Median E_2_ = 3,471 pmol/l. Maximal variation E_2_/12 h = 1,481 pmol/l.

The largest variation in P_4_ levels was seen in patients with median P_4_ > 250 nmol/l. In patient #2 with a median P_4_ of 283 nmol/l, P_4_ fluctuated from 293 nmol/l at 11 a.m. to 448 nmol/l at 12 p.m.—i.e., an increase of 155 nmol/l within 1 h. This fluctuation in P_4_ level was present even though s-LH was under the detection level throughout the day (Figure 2A). The increase in P_4_ was accompanied by a comparable increase in E_2_ (Figure 2A). Serum P_4_ concentrations during the 12-h period for that specific patient ranged from 183 nmol/l early in the morning to 448 nmol/l during the day—thus, a MAV during the study period of Δ265 nmol/l. In patient #6 (median P_4_ 376 nmol/l) and #3 (median P_4_ 277 nmol/l) a rapid elevation of P_4_ levels (Δ 70–75 nmol/l, respectively) was seen within a period of only 15 min without any concomitant LH activity (LH < 0.1 IU/l). In comparison, patient #7 had a median P_4_ of only 36 nmol/l and showed only minor fluctuations throughout the day with P_4_ levels ranging from 25 to 48 nmol/l following a small detectable increase in LH secretion (see Table 2 for complete daytime P_4_ values).

**Table 2:** Mid-luteal serum progesterone concentrations during daytime in 10 women undergoing *in vitro* fertilization treatment.

Patient											Median P_4_ (range)
**1**											
Time	8.00 a.m.	9.00 a.m.	9.15 a.m.	9.30 a.m.	9.45 a.m.	10.00 a.m.	11.00 a.m.	12.00 p.m.	13.00 p.m.	14.00 p.m.	
P_4_ nmol/l	126	106	91	93	88	97	87	93	99	107	
	15 p.m.	16 p.m.	17 p.m.	17.15 p.m.	17.30 p.m.	17.45 p.m.	18 p.m.	19 p.m.	20 p.m.		89 (63–126)
	102	89	69	72	78	78	73	67	63		ΔP_4_: 63

**2**											
Time	8.00 a.m.	9.00 a.m.	10.00 a.m.	10.15 a.m.	10.30 a.m.	10.45 a.m.	11.00 a.m.	12.00 p.m.	13.00 p.m.	14.00 p.m.	
P_4_ nmol/l	183	224	283	271	266	283	293	448	249	249	
	15.00 p.m.	15.15 p.m.	15.30 p.m.	15.45 p.m.	16.00 p.m.	17.00 p.m.	18.00 p.m.	19.00 p.m.	20.00 p.m.		283 (183–448)
	262	258	345	328	342	331	348	387	294		ΔP_4_: 265

**3**											
Time	6.00 a.m.	7.00 a.m.	7.15 a.m.	7.30 a.m.	7.45 a.m.	8.00 a.m.	9.00 a.m.	10.00 a.m.	11.00 a.m.	12.00 p.m.	
P_4_ nmol/l	370	320	323	301	272	347	281	244	235	301	
	12.15 p.m.	12.30 p.m.	12.45 p.m.	13.00 p.m.	14.00 p.m.	15.00 p.m.	16.00 p.m.	17.00 p.m.	18.00 p.m.		277 (225–370)
	283	269	277	278	230	225	251	255	232		ΔP_4_: 145

**4**											
Time	8.00 a.m.	8.15 a.m.	8.30 a.m.	8.45 a.m.	9.00 a.m.	10.00 a.m.	11.00 a.m.	12.00 p.m.	13.00 p.m.	14.00 p.m.	
P_4_ nmol/l	216	228	224	228	216	226	202	240	213	222	
	14.15 p.m.	14.30 p.m.	14.45 p.m.	15.00 p.m.	16.00 p.m.	17.00 p.m.	18.00 p.m.	19.00 p.m.	20.00 p.m.		213 (171–240)
	208	205	200	201	228	204	188	190	171		ΔP_4_: 69

**5**											
Time	9.00 a.m.	10.00 a.m.	11.00 a.m.	12.00 p.m.	13.00 p.m.	14.00 p.m.	15.00 p.m.	16.00 p.m.	17.00 p.m.	18.00 p.m.	
P_4_ nmol/l	131	122	110	119	136	97	92	89	70	77	
	19.00 p.m.	20.00 p.m.	21.00 p.m.								97 (70–136)
	70	96	71								ΔP_4_: 66

**6**											
Time	7.00 a.m.	8.00 a.m.	9.00 a.m.	10.00 a.m.	11.00 a.m.	11.15 a.m.	11.30 a.m.	11.45 a.m.	12.00 p.m.	13.00 p.m.	
P_4_ nmol/l	416	296	311	299	365	314	312	382	413	414	
	13.15 p.m.	13.30 p.m.	13.45 p.m.	14.00 p.m.	15.00 p.m.	16.00 p.m.	17.00 p.m.	18.00 p.m.	19.00 p.m.		376 (275–440)
	416	395	307	275	366	438	395	440	376		ΔP_4_: 165

**7**											
Time	7.00 a.m.	8.00 a.m.	9.00 a.m.	10.00 a.m.	11.00 a.m.	12.00 p.m.	13.00 p.m.	14.00 p.m.	15.00 p.m.	16.00 p.m.	
P_4_ nmol/l	27	25	32	36	40	39	36	34	29	29	
	17.00 p.m.	18.00 p.m.	19.00 p.m.								36 (25–48)
	39	48	37								ΔP_4_: 23

**8**											
Time	7.00 a.m.	8.00 a.m.	9.00 a.m.	9.15 a.m.	9.30 a.m.	9.45 a.m.	10.00 a.m.	11.00 a.m.	12.00 p.m.	13.00 p.m.	
P_4_ nmol/l	51	55	50	50	50	49	50	53	59	59	
	14.00 p.m.	15.00 p.m.	16.00 p.m.	16.15 p.m.	16.30 p.m.	16.45 p.m.	17.00 p.m.	18.00 p.m.	19.00 p.m.		55 (49–62)
	52	53	61	59	60	59	62	58	56		ΔP_4_: 13

**9**											
Time	7.00 a.m.	8.00 a.m.	9.00 a.m.	10.00 a.m.	11.00 a.m.	11.15 a.m.	11.30 a.m.	11.45 a.m.	12.00 p.m.	13.00 p.m.	
P_4_ nmol/l	65	64	63	58	52	52	50	51	51	59	
	14.00 p.m.	15.00 p.m.	16.00 p.m.	17.00 p.m.	18.00 p.m.	18.15 p.m.	18.30 p.m.	18.45 p.m.	19.00 p.m.		54 (51–65)
	54	58	55	58	56	53	53	53	52		ΔP_4_: 14

**10**											
Time	7.00 a.m.	8.00 a.m.	8.15 a.m.	8.30 a.m.	8.45 a.m.	9.00 a.m.	10.00 a.m.	10.15 a.m.	10.30 a.m.	10.45 a.m.	
P_4_ nmol/l	185	178	187	182	181	180	112	108	117	124	
	11.00 a.m.	12.00 p.m.	13.00 p.m.	14.00 p.m.	15.00 p.m.	16.00 p.m.	17.00 p.m.	18.00 p.m.	19.00 p.m.		161 (108–189)
	122	140	155	153	172	189	18	161	146		ΔP_4_: 81

*ΔP_4_ = individual maximum absolute variation in P_4_ during daytime = maximum P_4_ − minimum P_4_ concentration (nmol/l)*.

P_4_ SI conversion factor: nmol/l = 3.18*ng/ml.

**Figure 3:**
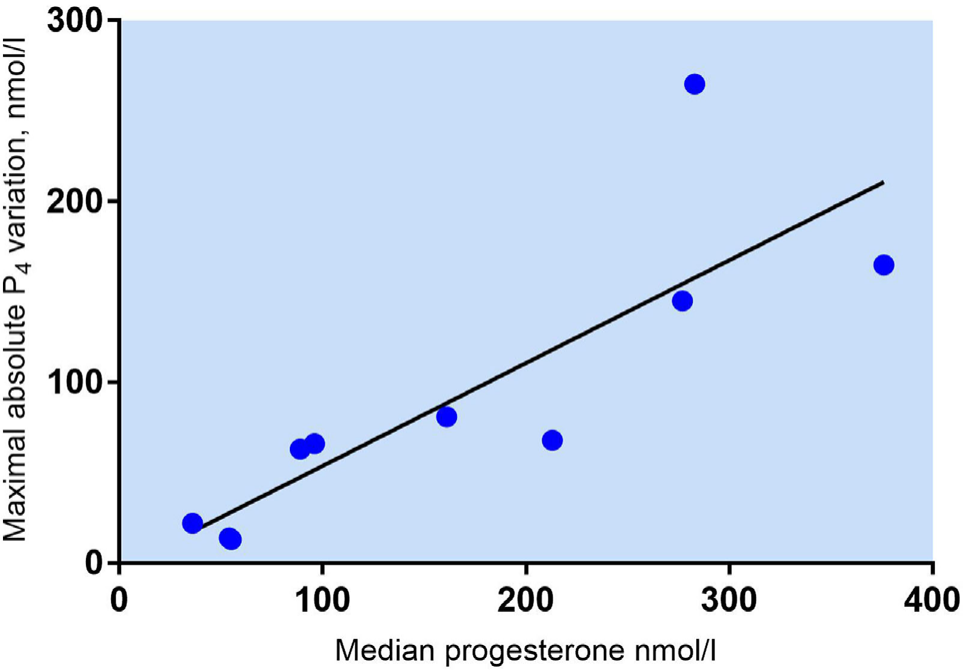
Maximum absolute variation in s-progesterone = P_4_ maximum − P_4_ minimum (nmol/l) in 10 patients undergoing *in vitro* fertilization treatment. Spearman’s *r* = 0.9273, *p* = 0.0001.

There was a positive correlation between median P_4_ levels and MAV in P_4_ during daytime (Spearman’s *r* = 0.9273, *p* = 0.0001). The magnitude of P_4_ pulses and thus the maximum variation is dependent on the median mid-luteal P_4_ concentration (Figure 3). In patients with median P_4_ > 250 nmol/l, very large fluctuations in serum P_4_ were seen during daytime with a median MAV of 165 nmol/l (range 145–265 nmol/l). Patients with median P_4_ between 89 and 213 nmol/l had median MAV of 68 nmol/l (range 63–81 nmol/l), whereas patients with very low mid-luteal P_4_ levels (median P_4_ < 60 nmol/l) had fairly constant serum P_4_ levels throughout the day (median MAV 14 nmol/l, range 13–23 nmol/l).

**Figure 4:**
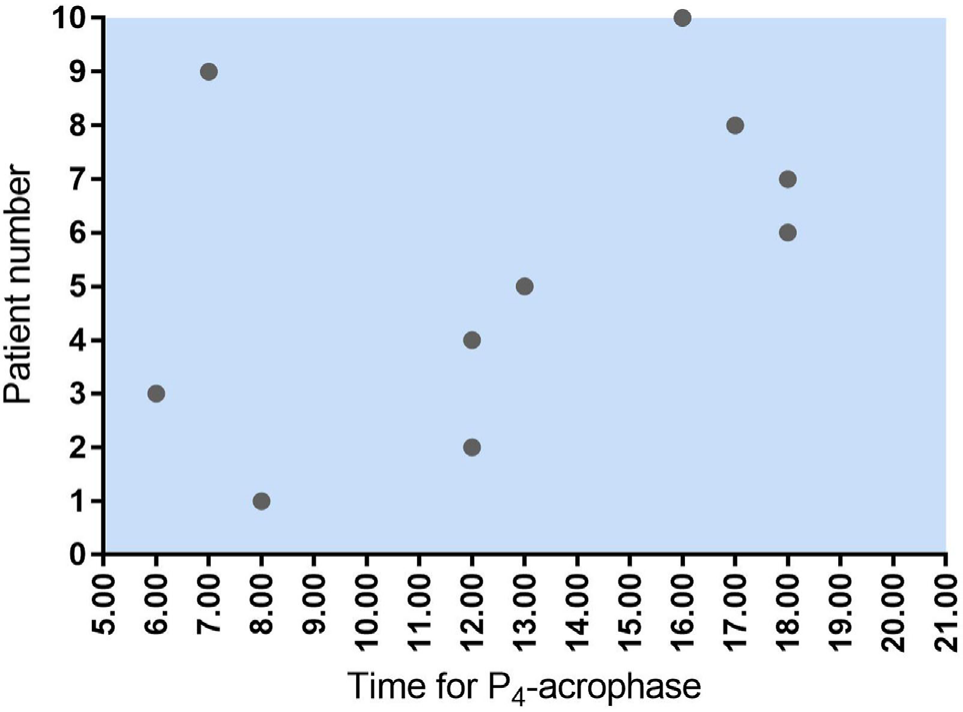
Time for maximum P_4_ concentration (acrophase) in 10 women undergoing IVF treatment. As seen, five women had their P_4_ acrophase before noon and five after noon. The same pattern was seen for P_4_ nadir.

There was no common general daytime rhythm for P_4_ in the 10 women examined, suggesting that the luteal phase is patient specific. Some patients had their highest hormone levels in the morning—others peaked during the day or in the early evening (see Figure 1). The time of P_4_ acrophase (zenith) and P_4_ nadir was before noon in half of the patients and after noon in the other half of patients (Figure 4).

### Daytime Variation in Serum Estradiol

Large fluctuations in mid-luteal serum E_2_ were also seen during the 12-h sampling time. In patient #2, E_2_ increased from 3,480 to 4,664 pmol/l in 1 h (Δ1,184 nmol/l) (Figure 2B). Patients had individual maximum E_2_ variations (Max E_2_ − Min E_2_) over 12 h ranging from Δ404 to Δ1,481 pmol/l. There was no correlation between median E_2_ levels and MAV in mid-luteal E_2_ (Spearman’s *r* = 0.4424, *p* = 0.20).

**Figure 5:**
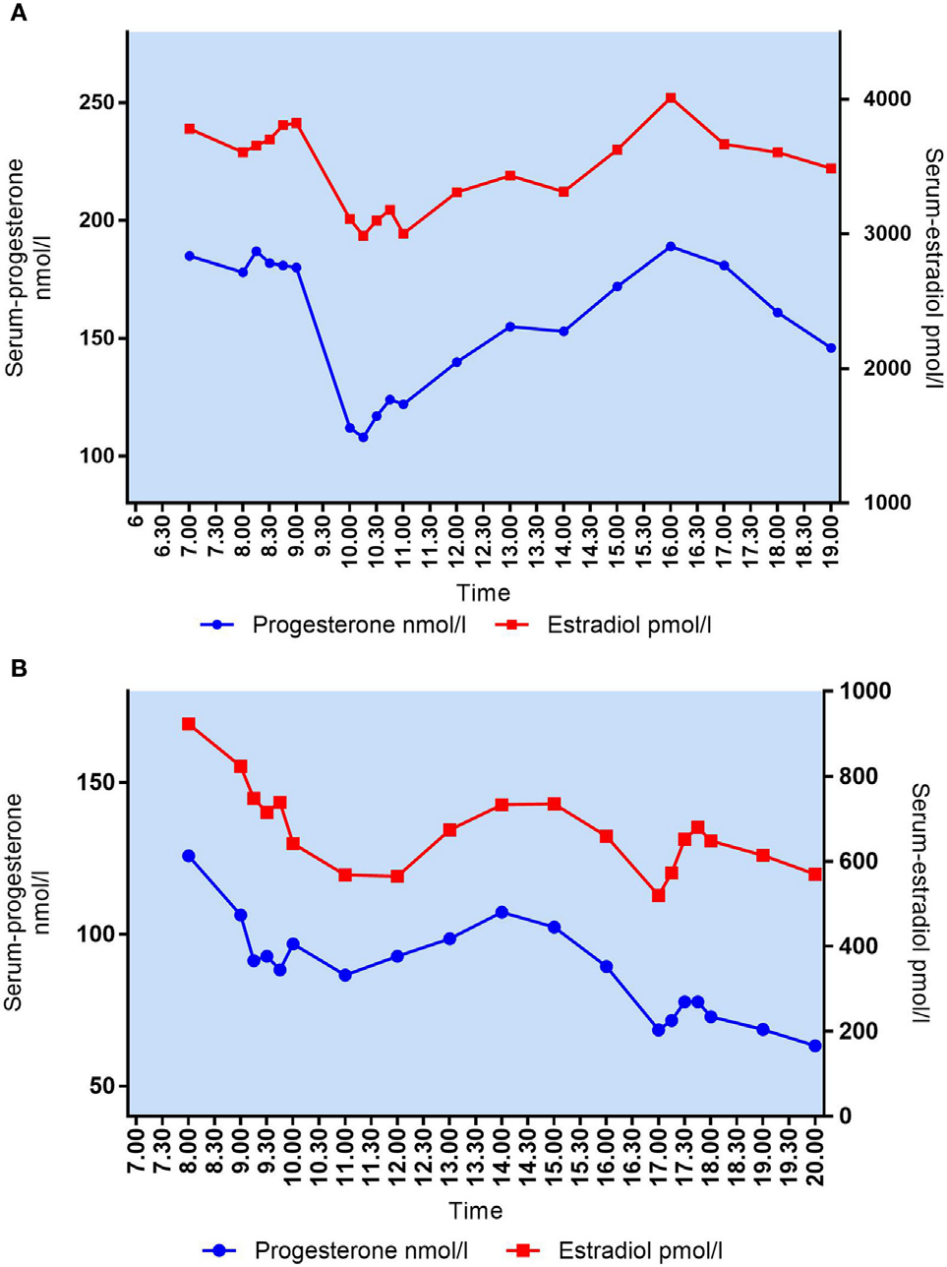
**(A)** Daytime variation in mid-luteal s-progesterone and s-estradiol in patient #10. Median P_4_ = 161 nmol/l, median E_2_ = 3,606 pmol/l. **(B)** Daytime variation in mid-luteal s-progesterone and s-estradiol in patient #1. Median P_4_ = 89 nmol/l, median E_2_ = 659 pmol/l.

As expected, P_4_ and E_2_ seem to be co-secreted from the CL showing similar patterns of fluctuations over time (Figure 5). Patients with median P_4_ < 60 nmol/l had E_2_ ranging from 541 to 1,552 pmol/l (median E_2_ 1,457 pmol/l) whereas patients with median P_4_ between 89 and 213 nmol/l had E_2_ levels from 659 to 4,884 pmol/l (median E_2_ 2,843 pmol/l). In patients with median P_4_ > 250 nmol/l, E_2_ ranged from 3,471 to 3,919 pmol/l (median E_2_ 3,874 pmol/l). There was a significant correlation between median P_4_ levels and median E_2_ levels during mid-luteal phase of the stimulated cycle (Spearman’s *r* = 0.8424, *p* = 0.002).

## Discussion

To the best of our knowledge, this is the first study to explore a possible daytime variation in P_4_ secretion during the mid-luteal phase in a group of women undergoing IVF treatment.

We found that the magnitude of mid-luteal P_4_ fluctuations following IVF treatment was dependent on the median P_4_ level. The largest P_4_ variations were seen in patients with median P_4_ exceeding 250 nmol/l (median MAV 165 nmol/l), whereas patients in the low P_4_ group (median P_4_ < 60 nmol/l) had relatively constant P_4_ levels throughout the day (median MAV 14 nmol/l). Patients showed a highly individual hormone secretion pattern without any obvious common daytime rhythm in P_4_ secretion. Serum E_2_ showed similar fluctuations in the mid-luteal phase with patients having individual E_2_ variations ranging from Δ404 to Δ1,481 pmol/l during the 12-h study time.

Earlier studies described the highly variable pattern of P_4_ secretion during the mid-luteal phase of naturally cycling women (15, 19, 20). These studies reported the presence of two distinguishable types of luteal P_4_ pulses—some preceded by a LH pulse and others non-concomitant to LH seen during time of pituitary quiescence. The latter seems to be the result of an autonomous P_4_ secretion from the CL, triggered and maintained by intraovarian concentrations of E_2_, oxytocin, and PGF_2α_ (21, 22). The CL consists of two types of steroidogenic cells, i.e., the small luteal cells (SLCs) derived from follicular theca cells and the large luteal cells (LLCs) originating from follicular granulosa cells. Both the small and the large cells have extensive capacity to produce P_4_. Moreover, both cells have unique steroidogenic functions and the “two-cell” mechanism of E_2_ biosynthesis appear to operate in the human CL analogous to the preovulatory follicle (23). Thus, the LLCs contain P450-aromatase essential for E_2_ synthesis whereas SLCs express P450c17 for androgen production (24, 25). Both types of luteal cells express E_2_ receptors (26) and E_2_ stimulation is a powerful trigger of P_4_ release from either cell type (27).

Isolation of large and SLCs from human corpora lutea has shown that once induced by the LH peak, the LLCs exhibit the greatest basal P_4_ production (28) and that this production is not increased by further LH stimulation (29). The LLCs produce P_4_ at a constant rate and are the dominant source of P_4_ during the early luteal phase (30). During this period, the P_4_ levels exhibit a non-pulsatile pattern both in the natural (15) and stimulated cycle (OPU + 2) (31). The responsivity of the SLCs to LH/hCG develops during the early mid-luteal phase where cells respond with a pronounced increase in P_4_ secretion in response to LH pulses. The luteal P_4_ contribution from the SLCs increase during the mid-and late luteal phase during which P_4_ secretion becomes highly pulsatile (32).

Thus, the endogenous mid-luteal P_4_ level consist of three parts—the basal P_4_ production from the LLCs, the P_4_ pulses from SLCs triggered by pituitary LH, and the autonomous P_4_ fluctuations independent of luteotrophic stimuli.

Previously, Wuttke et al. proposed a model to explain the LH-independent fluctuations in mid-luteal P_4_ levels based on autocrine and paracrine mechanisms in the luteal tissue (21). Upon stimulation with LH during the mid-luteal phase, the SLCs start secretion of P_4_ as well as androstenedione—the latter is subsequently converted to E_2_ in LLCs by P450-aromatase. The increased E_2_ concentration acts in an autocrine way in LLCs to increase the release of both P_4_ and oxytocin. Oxytocin stimulates fibroblasts to release PGF_2α_, which in turn stimulates further oxytocin as well as E_2_ secretion from the luteal cells. The isolated effect of oxytocin and PGF_2α_ upon the luteal cell lines is a decreased P_4_ secretion, but this effect is overridden by the concomitantly triggered increase in E_2_ which will elicit a pronounced P_4_ release. In this way, the LH pulse will stimulate an intra-luteal circuit involving auto-and paracrine effects of E_2_, oxytocin, PGF_2α_—and possible a variety of other regulatory peptides, i.e., Substance P—and the net effect is the generation of a P_4_ pulse. This circuit functions for hours without further gonadotropic support, thus generating several P_4_ pulses with gradually decreasing amplitude until the next LH pulse sets off the intra-luteal E_2_/P_4_ loop again. In contrast, in women with hypothalamic deficiency with suppressed LH levels and no LH pulses, mid-luteal P_4_ shows a non-pulsatile pattern, underlining the need for an initial high LH/hCG load to trigger the P_4_ circuit (21). The oxytocin induced P_4_ release can be prevented by treatment of the CL with tamoxifen—an estrogen receptor blocking agent—underlining the E_2_ regulation of the autonomous P_4_ pulses (27). This independent intra-luteal P_4_ pulse generator might serve as an additional biological safety mechanism preventing declining P_4_ levels in between LH pulses and might explain the function of the substantial E_2_ production during the luteal phase in humans.

In the stimulated IVF cycle, LH pulses are absent during the mid-luteal phase and serum LH levels are distinctly suppressed (33). The hCG bolus administered for ovulation induction or as luteal phase support exerts a tonic and constant stimulation on the luteal tissue due to the prolonged half-life of hCG and, therefore, cannot account for the rapid P_4_ fluctuations seen during the mid-luteal phase in this study. The standard vaginal P_4_ supplementation reaches steady state during the early luteal phase and contributes with remarkably constant serum P_4_ levels though out the day despite multiple daily vaginal doses (34). The very large fluctuations in serum P_4_ seen in the present study are, therefore, likely to be the result of the autonomous intraovarian P_4_ circuit. This is further emphasized by the fact that P_4_ peaks are accompanied by concomitant E_2_ rises and exogenous E_2_ was not provided as part of the luteal phase support.

We were not able to detect a common general pattern of P_4_ secretion during daytime in the 10 patients examined. The peak and nadir P_4_ levels occurred at different times in different patients, and the course of hormone levels during the day showed highly individual rhythms. This is in agreement with studies performed during the mid-luteal phase of the natural cycle (3, 19). In a study of seven women studied over 24 h in the mid-luteal phase of the natural cycle, the P_4_ acrophase varied from 10.31 a.m. to 11.33 p.m. (16). Based on the lack of a diurnal reproducible pattern for mid-luteal P_4_ in the IVF cycle the accuracy of the P_4_ measurement is not improved by a fixed timing of blood sampling and, thus, the P_4_ measurement could be performed at any time during clinic opening hours.

During the natural cycle both late follicular E_2_ levels, follicular diameter at the time of ovulation as well as area under the LH surge curve correlate poorly to the subsequent luteal phase P_4_ level (3). Thus, predicting patients with insufficient luteal P_4_ levels is troublesome based on the follicular development as abnormal luteal phases can be seen in cycles characterized by normal folliculogenesis (35). In the present study, the two patients with the lowest P_4_ levels (36 and 55 nmol/l) had 17 and 19 follicles, respectively, on the day of OPU, showing that a large number of CLs do not warrant a high P_4_ output in the luteal phase. For this reason monitoring of luteal phase P_4_ could be of value to detect patients with low P_4_ levels, who might benefit from additional exogenous P_4_ therapy. However, the prerequisite for easy luteal phase monitoring is that the validity of a single measured P_4_ value is reliable and gives a reasonable estimate of the CL capacity of the patient.

We acknowledge that the small sample size of this study may limit the validity of general interpretations. However, we consider this explorative preliminary study to be pioneering as part of basic research and, importantly, it is the first to explore the mid-luteal P_4_ fluctuations in different types of IVF cycles. The autonomous LH-independent P_4_ bursts from the ovaries during the mid-luteal phase were seen in both GnRH analog types (GnRH antagonist and long GnRHa protocol) as well as after different types of triggering of final oocyte maturation (hCG or GnRH agonist). Thus, it seems that these autonomous episodic luteal P_4_ peaks are generated independently of the choice of treatment regimen and may, therefore, also apply to other IVF stimulation protocols.

## Conclusion

Based on the 10 women examined in this study, we state that the accuracy of a single mid-luteal serum progesterone measurement as an approximation of mean P_4_ levels throughout the day depends on the P_4_ concentration and that women with low P_4_ levels (P_4_ < 60 nmol/l) exhibit clinically stable P_4_ levels during daytime. Thus, a single P_4_ measurement in the low progesterone patient reflects quite accurately the CL function and a measured low P_4_ value can, therefore, be regarded as a “true low value.” Future studies should clarify, whether additional exogenous P_4_ support administered to the low luteal P_4_ patient group can improve the reproductive outcome.

## Data Availability

The raw data supporting the conclusion of this manuscript will be made available by the authors, without undue reservation, to any qualified researcher.

## Ethics Statement

The study was conducted according to the declaration of Helsinki for Medical Research and approved by the local Ethics Committee of the Central Denmark Region. All patients provided written informed consent to participate in the study.

## Author Contributions

LT designed and conducted the study. LT drafted the manuscript and UK, CA, and PH all contributed to the interpretation of data and critically reviewed the manuscript. All coauthors accepted the final draft.

## Conflict of Interest Statement

LT received an unrestricted research grant from Ferring Pharmaceuticals outside of this work. PH received unrestricted research grants from MSD, Merck, and Ferring Pharmaceuticals as well as honoraria for lectures from MSD, Merck, and Finox outside of this work. UK received honoraria for lectures from MSD and Ferring Pharmaceuticals outside of this work. CA received unrestricted research grants from MSD, IBSA, and Ferring Pharmaceuticals as well as honoraria for lectures from MSD and IBSA outside of this work.

## References

[1] Wiltbank MC Salih SM Atli MO Luo W Bormann CL Ottobre JS Comparison of endocrine and cellular mechanisms regulating the corpus luteum of primates and ruminants. Anim Reprod (2012) 9(3):242–59. 23750179 PMC3674567

[2] Rossmanith WG Laughlin GA Mortola JF Yen SS. Secretory dynamics of oestradiol (E2) and progesterone (P4) during periods of relative pituitary LH quiescence in the midluteal phase of the menstrual cycle. Clin Endocrinol (Oxf) (1990) 32(1):13–23. 10.1111/j.1365-2265.1990.tb03745.x 2331809

[3] Soules MR Clifton DK Steiner RA Cohen NL Bremner WJ. The corpus luteum: determinants of progesterone secretion in the normal menstrual cycle. Obstet Gynecol (1988) 71(5):659–66. 3357651

[4] Humaidan P Bungum L Bungum M Hald F Agerholm I Blaabjerg J Reproductive outcomes using a GnRH antagonist (Cetrorelix) for luteolysis and follicular synchronization in poor responder IVF/ICSI patients treated with a flexible GnRH antagonist protocol. Reprod Biomed Online (2005) 11(6):679–84. 10.1016/S1472-6483(10)61685-9 16417730

[5] Humaidan P Ejdrup Bredkjaer H Westergaard LG Yding Andersen C. 1,500 IU human chorionic gonadotropin administered at oocyte retrieval rescues the luteal phase when gonadotropin-releasing hormone agonist is used for ovulation induction: a prospective, randomized, controlled study. Fertil Steril (2010) 93(3):847–54. 10.1016/j.fertnstert.2008.12.042 19200959

[6] Yding Andersen C Vilbour Andersen K. Improving the luteal phase after ovarian stimulation: reviewing new options. Reprod Biomed Online (2014) 28(5):552–9. 10.1016/j.rbmo.2014.01.012 24656557

[7] Blake EJ Norris PM Dorfman SF Longstreth J Yankov VI. Single and multidose pharmacokinetic study of a vaginal micronized progesterone insert (Endometrin) compared with vaginal gel in healthy reproductive-aged female subjects. Fertil Steril (2010) 94(4):1296–301. 10.1016/j.fertnstert.2009.06.014 19608168

[8] Kisicki JC Pharmacokinetic Study of Three Dosage Strengths of COL-1620 with Natural Progesterone. Columbia Research Laboratories Data on file. Crinone Product Monograph (2008). Avaliable from: https://dailymed.nlm.nih.gov/dailymed/drugInfo.cfm?id=13472

[9] Colburn WA Pharmacokinetic Study of 90 mg Strength of COL-1620 with Natural Progesterone, B.I.D. Columbia Research Laboratories Data on file. Crinone Product Monograph (2008). Avaliable from: https://dailymed.nlm.nih.gov/dailymed/drugInfo.cfm?id=13472

[10] Humaidan P Polyzos NP Alsbjerg B Erb K Mikkelsen AL Elbaek HO GnRHa trigger and individualized luteal phase hCG support according to ovarian response to stimulation: two prospective randomized controlled multi-centre studies in IVF patients. Hum Reprod (2013) 28(9):2511–21. 10.1093/humrep/det249 23753114

[11] Niemann H Sacher B Elsaesser F. Pregnancy rates relative to recipient plasma progesterone levels on the day of nonsurgical transfer of frozen/thawed bovine embryos. Theriogenology (1985) 23(4):631–9. 10.1016/0093-691X(85)90197-9 16726032

[12] Nogueira MF Melo DS Carvalho LM Fuck EJ Trinca LA Barros CM. Do high progesterone concentrations decrease pregnancy rates in embryo recipients synchronized with PGF2alpha and eCG? Theriogenology (2004) 61(7–8):1283–90. 10.1016/j.theriogenology.2003.07.012 15036962

[13] Yovich JL Conceicao JL Stanger JD Hinchliffe PM Keane KN. Mid-luteal serum progesterone concentrations govern implantation rates for cryopreserved embryo transfers conducted under hormone replacement. Reprod Biomed Online (2015) 31(2):180–91. 10.1016/j.rbmo.2015.05.005 26099447

[14] Labarta E Mariani G Holtmann N Celada P Remohi J Bosch E. Low serum progesterone on the day of embryo transfer is associated with a diminished ongoing pregnancy rate in oocyte donation cycles after artificial endometrial preparation: a prospective study. Hum Reprod (2017) 32(12):2437–42. 10.1093/humrep/dex316 29040638

[15] Filicori M Butler JP Crowley WF Jr. Neuroendocrine regulation of the corpus luteum in the human. Evidence for pulsatile progesterone secretion. J Clin Invest (1984) 73(6):1638–47. 10.1172/JCI111370 6427277 PMC437074

[16] Veldhuis JD Christiansen E Evans WS Kolp LA Rogol AD Johnson ML. Physiological profiles of episodic progesterone release during the midluteal phase of the human menstrual cycle: analysis of circadian and ultradian rhythms, discrete pulse properties, and correlations with simultaneous luteinizing hormone release. J Clin Endocrinol Metab (1988) 66(2):414–21. 10.1210/jcem-66-2-414 3339114

[17] Reame N Sauder SE Kelch RP Marshall JC. Pulsatile gonadotropin secretion during the human menstrual cycle: evidence for altered frequency of gonadotropin-releasing hormone secretion. J Clin Endocrinol Metab (1984) 59(2):328–37. 10.1210/jcem-59-2-328 6429184

[18] Tavaniotou A Albano C Smitz J Devroey P. Comparison of LH concentrations in the early and mid-luteal phase in IVF cycles after treatment with HMG alone or in association with the GnRH antagonist Cetrorelix. Hum Reprod (2001) 16(4):663–7. 10.1093/humrep/16.4.663 11278214

[19] Fujimoto VY Clifton DK Cohen NL Soules MR. Variability of serum prolactin and progesterone levels in normal women: the relevance of single hormone measurements in the clinical setting. Obstet Gynecol (1990) 76(1):71–8. 2359568

[20] Rossmanith WG Laughlin GA Mortola JF Johnson ML Veldhuis JD Yen SS. Pulsatile cosecretion of estradiol and progesterone by the midluteal phase corpus luteum: temporal link to luteinizing hormone pulses. J Clin Endocrinol Metab (1990) 70(4):990–5. 10.1210/jcem-70-4-990 2318954

[21] Wuttke W Theiling K Hinney B Pitzel L. Regulation of steroid production and its function within the corpus luteum. Steroids (1998) 63(5–6):299–305. 10.1016/S0039-128X(98)00037-3 9618790

[22] Bah MM Acosta TJ Pilawski W Deptula K Okuda K Skarzynski DJ. Role of intraluteal prostaglandin F(2alpha), progesterone and oxytocin in basal and pulsatile progesterone release from developing bovine corpus luteum. Prostaglandins Other Lipid Mediat (2006) 79(3–4):218–29. 10.1016/j.prostaglandins.2006.01.002 16647636

[23] Fisch B Margara RA Winston RM Hillier SG. Cellular basis of luteal steroidogenesis in the human ovary. J Endocrinol (1989) 122(1):303–11. 10.1677/joe.0.1220303 2769154

[24] Maybin JA Duncan WC. The human corpus luteum: which cells have progesterone receptors? Reproduction (2004) 128(4):423–31. 10.1530/rep.1.00051 15454637

[25] Devoto L Fuentes A Kohen P Cespedes P Palomino A Pommer R The human corpus luteum: life cycle and function in natural cycles. Fertil Steril (2009) 92(3):1067–79. 10.1016/j.fertnstert.2008.07.1745 18793774

[26] van den Driesche S Smith VM Myers M Duncan WC. Expression and regulation of oestrogen receptors in the human corpus luteum. Reproduction (2008) 135(4):509–17. 10.1530/REP-07-0427 18367511

[27] Maas S Jarry H Teichmann A Rath W Kuhn W Wuttke W. Paracrine actions of oxytocin, prostaglandin F2 alpha, and estradiol within the human corpus luteum. J Clin Endocrinol Metab (1992) 74(2):306–12. 10.1210/jc.74.2.306 1730809

[28] Friden BE Hagstrom H Lindblom B Sjoblom P Wallin A Brannstrom M Cell characteristics and function of two enriched fraction of human luteal cells prolonged culture. Mol Hum Reprod (1999) 5(8):714–9. 10.1093/molehr/5.8.714 10421797

[29] Ohara A Mori T Taii S Ban C Narimoto K. Functional differentiation in steroidogenesis of two types of luteal cells isolated from mature human corpora lutea of menstrual cycle. J Clin Endocrinol Metab (1987) 65(6):1192–200. 10.1210/jcem-65-6-1192 3119652

[30] Jones GS Corpus luteum: composition and function. Fertil Steril (1990) 54(1):21–6. 10.1016/S0015-0282(16)53630-9 2192920

[31] Tannus S Burke Y McCartney CR Kol S. GnRH-agonist triggering for final oocyte maturation in GnRH-antagonist IVF cycles induces decreased LH pulse rate and amplitude in early luteal phase: a possible luteolysis mechanism. Gynecol Endocrinol (2017) 5:1–5. 10.1080/09513590.2017.1318275 28440715

[32] Jones GS. Luteal phase defect: a review of pathophysiology. Curr Opin Obstet Gynecol (1991) 3(5):641–8. 10.1097/00001703-199110000-00003 1958796

[33] Wolfram J Siegberg R Apter D Alfthan H Stenman UH Laatikainen T. Pulsatility of serum-luteinizing hormone during hyperstimulation with clomiphene citrate and human menopausal gonadotropin for in vitro fertilization. Fertil Steril (1989) 52(5):817–20. 10.1016/S0015-0282(16)53045-3 2509253

[34] Devroey P Palermo G Bourgain C Van Waesberghe L Smitz J Van Steirteghem AC. Progesterone administration in patients with absent ovaries. Int J Fertil (1989) 34(3):188–93. 2567713

[35] Grunfeld L Sandler B Fox J Boyd C Kaplan P Navot D. Luteal phase deficiency after completely normal follicular and periovulatory phases. Fertil Steril (1989) 52(6):919–23. 10.1016/S0015-0282(16)53152-5 2591570

